# Neurotoxicity and Mechanism in Zebrafish Embryo Induced by Tetrabromobisphenol A bis (2-Hydroxyethyl) Ether (TBBPA-DHEE) Exposure

**DOI:** 10.3390/toxics13020076

**Published:** 2025-01-22

**Authors:** Xinyu Zhang, Liguo Guo, Yiwen Luo, Xia Xu, Ying Han, Hui Chen, Haohao Sun, Yingang Xue, Guixiang Ji

**Affiliations:** 1 School of Environmental Science and Engineering, Changzhou University, Changzhou 213164, China; xinyuzhang_0505@163.com (X.Z.); harriethan@yeah.net (Y.H.); huichen@cczu.edu.cn (H.C.); shh_cz@163.com (H.S.); 2 Nanjing Institute of Environmental Sciences, Ministry of Ecology and Environment, Nanjing 210042, China; guoliguo@nies.org (L.G.); luoyiwen@nies.org (Y.L.); 3 College of Urban Construction, Changzhou University, Changzhou 213164, China; xuxia@cczu.edu.cn

**Keywords:** neurotoxicity, zebrafish, TBBPA-DHEE, oxidative stress, transcriptome

## Abstract

Tetrabromobisphenol A bis (2-hydroxyethyl) ether (TBBPA-DHEE), a derivative of TBBPA, has been frequently detected in the environment. In this study, the median lethal concentration (LC_50_) of TBBPA-DHEE at 96 h post-fertilization (hpf) was 1.573 mg/L. Based on the reported environmental concentrations, we investigated the effects of TBBPA-DHEE on the nervous system of zebrafish embryos following exposure to varying concentrations (0, 20, 100, and 500 μg/L) for 4 to 144 hpf. Our results indicated that exposure to 100 μg/L at 144 hpf led to behavioral abnormalities in zebrafish. Furthermore, exposure to TBBPA-DHEE inhibited the development of the central nervous system and motor neurons in zebrafish. Real-time polymerase chain reaction (PCR) analysis revealed that exposure to TBBPA-DHEE significantly downregulated the expression levels of neurodevelopmental genes (*shha*, *syn2a*, *elavl3*, *gfap*, and *gap43*). Additionally, TBBPA-DHEE increased oxidative stress in zebrafish. Transcriptomic analysis demonstrated that exposure to TBBPA-DHEE affected the signaling pathways involved in neurodevelopment. Overall, this study demonstrated that TBBPA-DHEE may disrupt the early development of the nervous system, leading to abnormal motor behavior in zebrafish larvae, and provided novel insights into the potential mechanisms of TBBPA-DHEE neurotoxicity.

## 1. Introduction

Tetrabromobisphenol A (TBBPA) is a flame retardant commonly found in various products and environmental media and poses significant health risks [1,2]. TBBPA has been shown to disrupt the endocrine system [3], cause reproductive [4], neurological [5], and hepatic toxicity [6], and may be transmitted to infants through breast milk and the placenta [7,8]. Due to the numerous hazards of TBBPA, several derivatives with similar structures have been developed, such as TBBPA bis (2-hydroxyethyl) ether (TBBPA-DHEE), TBBPA bis (2,3-dibromopyl) ether (TBBPA-DBPE), and TBBPA diallyl ether (TBBPA-DAE), and their use is increasing [9].

TBBPA-DHEE, a derivative flame retardant of TBBPA, is widely used in electronic products, including circuit boards, computers, and mobile phones [10]. With its widespread use, TBBPA-DHEE has been detected in various environmental media. In China, the concentration of TBBPA-DHEE in water samples from a tributary of the Bohai Sea is 250 ng/L, which represents the highest detected concentration of TBBPA derivative species in this water body [11]. The concentration of TBBPA-DHEE detected in surface water near an electronic waste recycling zone is 1.33 ng/mL [12]. Furthermore, the accumulated concentration of TBBPA-DHEE detected in aquatic organisms in this region is 2.66 ng/g [12]. Existing research has indicated that TBBPA-DHEE demonstrated similar endocrine-disrupting effects, cytotoxicity, and reproductive toxicity to TBBPA [13,14,15]. However, a systematic investigation into the neurotoxic effects and mechanism of TBBPA-DHEE on aquatic organisms has not yet been conducted.

Among the derivatives of TBBPA, TBBPA-DHEE exhibits a higher endocrine-disrupting effect and shows greater cytotoxicity and reproductive toxicity than other derivatives [14,16,17]. Liu et al. found that TBBPA-DHEE induces PC12 cell death by inducing oxidative stress and activating caspase activity [13]. Liu et al.’s histopathological study on neonatal rats after nasal administration of TBBPA-DHEE showed that TBBPA-DHEE cause the proliferation and swelling of nerve cells, thereby affecting the basic cellular functions and neural processes in the rat brain [14]. Research on the sexual development of zebrafish revealed that exposure to TBBPA-DHEE causes hormonal disorders related to sexual development and reduces the gene transcription level of key pathways for reproductive development [15]. TBBPA-DHEE has been detected in large quantities in the water environment and exhibits similar toxic effects to TBBPA. Its ecological risk has attracted significant attention. However, systematic studies on the early neurotoxic effects and mechanisms of TBBPA-DHEE in aquatic organisms have not yet been conducted.

Zebrafish serve as a prevalent model for assessing the impacts and toxicity of contaminants on aquatic animals, offering benefits such as diminutive size, short developmental cycle, and strong fecundity [18]. The nervous system of zebrafish develops during the period from 6 h to 144 h [19,20], and its embryos are transparent, enabling the visualization of the neural development processes and neural activities [21]. Consequently, zebrafish are commonly employed to evaluate the neurotoxicity of pollutants and to elucidate the mechanisms of neurodevelopmental disorders.

This study aimed to further explore the neurotoxicity of TBBPA-DHEE and its potential molecular mechanisms using zebrafish models, including *Tg (Hb9: eGFP)* and *Tg (Gad1b: mCherry)* transgenic zebrafish, combined with motor behavior experiments, oxidative stress level assessment, and transcriptome analysis. Our results will elucidate the environmental hazards of TBBPA-DHEE and provide a reference for the action mode of TBBPA on the nervous system.

## 2. Materials and Methods

### 2.1. Chemicals and Reagents

TBBPA-DHEE (CAS No. 4162-45-2, >93% purity) was purchased from Shanghai Macklin Biochemical Technology Co., Ltd. (Shanghai, China) Dimethyl sulfoxide (DMSO, CAS No. 67-68-5) was acquired from Beijing Solarbio Science & Technology Co., Ltd. (Beijing, China) All chemical reagents and solvents employed in the investigation were of analytical grade. SYBR-green RT-PCR and reverse transcription kits were acquired from Vazyme Biotech Co., Ltd. (Nanjing, China) ROS Assay Kit, Lipid Peroxidation malondialdehyde (MDA) Assay Kit, Total Superoxide Dismutase (SOD) Assay Kit with WST-8 and Catalase (CAT) Assay Kit were purchased from Beyotime Biotech Co., Ltd. (Nanjing, China).

### 2.2. Zebrafish Maintenance and Embryo Collection

The AB wild type (WT) and two strains of transgenic zebrafish (*Hb9: eGFP* and *Gad1b: mCherry*) were purchased from the Institute of Hydrobiology, Chinese Academy of Sciences. This work examines two transgenic zebrafish lines, *Tg (Hb9: eGFP)* and *Tg (Gad1b: mCherry)*, which specifically express green fluorescent protein in motor neuron axons and red fluorescence in GAD67-positive GABAergic neurons, respectively. All zebrafish were reared in one aquaculture system, in which the temperature of the circulating water was maintained at 28 ± 0.5 °C, the conductivity was kept within the range of 500–600 μs/cm and pH at about 7.0–8.0. The light-dark cycle in the breeding room was set at 14 h:10 h. The zebrafish were fed three meals of freshly harvested Artemia nauplii every day.

On the eve of the exposure experiment, wild-type zebrafish were relocated to trapezoidal spawning boxes at a male-to-female ratio of 1:2 and retained overnight, with a baffle partitioning the males and females. Early the following morning, the baffle was removed and the illumination was activated to induce spawning behavior. It could be observed that the males began to chase the females. After 30–40 min, the embryos were harvested, and viable fertilized embryos were identified using a stereomicroscope (Nikon SMZ25, Okinawa, Japan) for use. All procedures using zebrafish received approval from the Animal Ethics Committee of the Nanjing Institute of Environmental Sciences (the license number is IACUC-20240226) and were carried out in accordance with relevant guidelines and regulations.

### 2.3. Embryos Treatment and Morphological Observation

The TBBPA-DHEE stock solution, which was dissolved in DMSO at a concentration of 10 mg/mL, was serially diluted with fish water to obtain the working concentrations. The Fish Embryo Acute Toxicity (FET) Test was conducted according to the guideline OECD Test No. 236 to determine the acute toxicity of TBBPA-DHEE to zebrafish embryos. Based on the results of the FET test (LC_50_ = 1.573 mg/L), the concentration of 1/3, 1/15 and 1/100 96 h LC_50_ (20, 100, and 500 μg/L) were selected for the following toxicity studies.

The embryos were mixed and randomly allocated in petri dishes with 50 mL of TBBPA-DHEE at varying concentrations. During the trial period, 50% of the exposure solution was replaced every 24 h. The times of spontaneous movements and the area of the yolk sac of the embryos at 24 hpf were recorded. The hatching rate was recorded at 48 hpf and 72 hpf. Heart rate, body length, and eye area were assessed at 72 hpf, whereas body length, eye area, and swim bladder area were measured at 144 hpf. After immobilizing the zebrafish larvae on a confocal dish using 4% methylcellulose, the phenotypes of the target organs in the transgenic zebrafish were measured utilizing a fluorescent stereomicroscope (Nikon, SMZ225, Japan). Each experiment was conducted three times.

### 2.4. Motor Behavior Evaluation

At 144 hpf, the zebrafish larvae, comprising the control and each exposure group, were placed into a 24-well plate, with 1 mL of exposure solution and one larva in each well. After 5 min of adaptation in darkness, the Danio Vision system (Noldus, Wageningen, The Netherlands) was employed to record the movement behavior of the zebrafish larvae under alternating light and dark conditions (5 min of light–5 min of darkness–5 min of light–5 min of darkness). The data of the distance, time, average distance, and average speed of their movement behavior as well as the swimming trajectory map were recorded using the EthoVision XT software (Vision 16; Noldus). After eliminating three outliers that significantly deviated from the overall distribution trend, the data of nine larvae in each concentration group were retained.

### 2.5. RNA Extraction and Real-Time Quantitative PCR

Following the approach of Gu J. et al. [22], at 144 hpf, 30 larvae were collected from each group, and total RNA was extracted using TRIzol reagent (Takara, Kusatsu, Japan). The RNA concentration was evaluated using a Nanodrop 2000 spectrophotometer (Thermo Fisher Scientific, Waltham, MA, USA). HiScript ||| RT SuperMix for qPCR (+gDNA wiper) (Vazyme, Nanjing, China) was used to synthesize the first-strand cDNA, and ChamQ Universal SYBR qPCR Master Mix (Vazyme, Nanjing, China) was used to prepare the qPCR system. Gene expression profiles were assessed with quantitative real-time PCR utilizing the CFX ConnectTM real-time system (Bio-Rad, Hercules, CA, USA). The amplification system contained 10 μL of SYBR-green premix, 0.4 μL of each primer, 1 μL of cDNA template, and 8.2 μL of ddH2O. The PCR program was as follows: initial denaturation at 95 °C for 30 s, followed by 40 cycles of denaturation at 95 °C for 10 s, and annealing/extension at 60 °C for 30 s. The relative expression was calculated by the 2^−△△Ct^ method, where Ct is the average threshold cycle value. The primers utilized in this investigation were specifically constructed and validated by BLAST, and Table S1 contains the list of primer sequences. *β-actin* served as the internal control gene, and PCR primers (*shha*, *syn2a*, *elavl3*, *gfap* and *gap43*) were purchased from Generay Biotech (Shanghai, China). Each sample was subjected to three biological replicates and three technological replicates to verify data reliability and comparability.

### 2.6. Oxidative Stress Analysis

At 144 hpf, nine larvae were aspirated from each group, stained with the 2′,7′-Dichlorodihydrofluorescein Diacetate (DCFH-DA) probe, and incubated at 28 °C for 30 min. Following three rinses with fish water, the samples were photographed using a fluorescence stereomicroscope (Nikon, SMZ225, Okinawa, Japan), and the fluorescence intensity was quantified using ImageJ. After 144 hpf exposure to TBBPA-DHEE, the zebrafish were harvested into Eppendorf tubes containing PBS (0.1 M, pH 8.0), homogenized, and centrifuged, and the supernatants were collected for subsequent analysis. Following the determination of protein concentration via the BCA Protein Assay Kit (Biosharp, Hefei, China), the activities of CAT and SOD, as well as the MDA content, were assessed in accordance with the respective kit instructions, with all results were normalized according to the protein concentration. Each experiment was repeated three times.

### 2.7. Transcriptome Analysis

The transcriptomics was performed to investigate the underlying toxic mechanism of TBBPA-DHEE. Briefly, the Total RNA was extracted from zebrafish larval tissue samples of control and high treatment groups (500 μg/L) and then sequenced by cluster profile software package. For these differentially expressed genes (DEGs, relative to the control) that were observed after exposure to TBBPA-DHEE, we performed gene ontology (GO) clustering and KEGG enrichment analysis using DAVID (Database for Annotation, Visualization and Integrated Discovery) Bioinformatics Resources 6.8.

### 2.8. Statistical Analysis

All statistical analyses were performed using SPSS 27.0 software and GraphPad Prime 8.0 software, and results are expressed as mean ± SEM. The substantial difference was evaluated using a one-way ANOVA test, followed by Dunnett’s multiple comparisons tests (* *p* < 0.05, ** *p* < 0.01 and *** *p* < 0.001).

## 3. Results

### 3.1. Developmental Toxicity of TBBPA-DHEE in Zebrafish Embryos and Larvae

**Figure 1:**
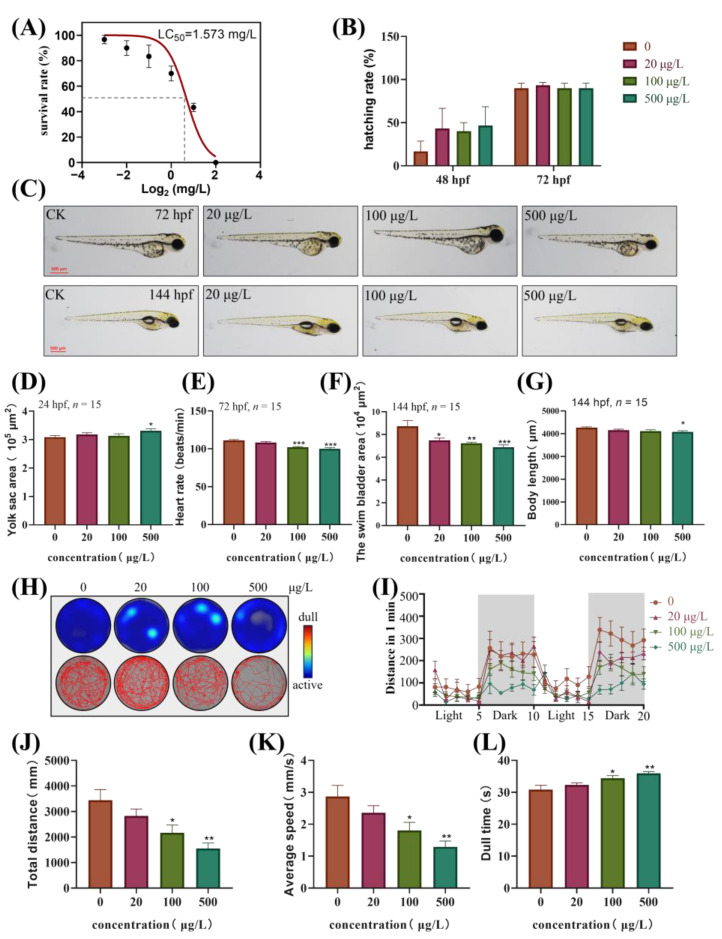
Effects of TBBPA-DHEE on the zebrafish embryos and larvae’s development index. (**A**) Fitted curves of survival of zebrafish larvae exposure to TBBPA-DHEE at 96 hpf at concentration gradients (0.125 mg/L, 0.25 mg/L, 0.5 mg/L, 1 mg/L, 2 mg/L, 4 mg/L); (**B**) The zebrafish larvae hatching rate following TBBPA-DHEE exposure at 48 and 72 hpf; (**C**) The typical figure of zebrafish larvae at 72 and 144 hpf; (**D**) The yolk sac area statistical chart of zebrafish embryo at 24 hpf; (**E**) The heart rate statistical chart of zebrafish larvae at 72 hpf; (**F**,**G**) The statistical chart of bladder size and body length at 144hpf; (**H**–**L**) The exemplary locomotion tracks, distance traveled per minute, total distance, average speed and dull time after TBBPA-DHEE exposure in 144 hpf larva zebrafish. (* *p* < 0.05, ** *p* < 0.01 and *** *p* < 0.001).

Embryos of zebrafish were exposed to TBBPA-DHEE in a concentration gradient (0.125, 0.25, 0.5, 1, 2 and 4 mg/L) for 24, 48, 72, and 96 h to determine survival. The results showed that TBBPA-DHEE caused embryonic mortality in a concentration-dependent manner. After 96 h, the LC_50_ value for TBBPA-DHEE was 1.573 mg/L (Figure 1A).

Consequently, the gradient concentrations of TBBPA-DHEE (0 μg/L, 20 μg/L, 100 μg/L, and 500 μg/L) were used in subsequent experiments. There was no significant change in hatchability in the exposed group compared to the control group (Figure 1B). However, the yolk sac area of embryos exposed to 500 μg/L significantly increased at 24 hpf (Figure 1D, *p* < 0.05). Additionally, the heart rate of zebrafish exposed to 100 and 500 μg/L was significantly lower than that of the control group at 72 hpf (Figure 1E, *p* < 0.001). Furthermore, after six days of TBBPA-DHEE exposure, a dose-dependent reduction in swim bladder area was observed in all groups (Figure 1F, *p* < 0.001), and a significant reduction in body length in the 500 μg/L group (Figure 1G, *p* < 0.05).

### 3.2. The Motor Ability of Zebrafish Larvae is Inhibited After Exposure to TBBPA-DHEE

For the analysis of behavioral and locomotor abilities of zebrafish larvae at 144 hpf, as shown in Figure 1, the experimental results indicated that the total movement distance and average swimming speed of zebrafish larvae exposed to 100 and 500 μg/L significantly decreased (Figure 1J,K, *p* < 0.05), to 45.01% and 62.88% of the control group, respectively. Meanwhile, the larvae in the high-concentration groups demonstrated diminished responsiveness to the transition between light and dark environments in comparison to the control group (Figure 1H,I). Additionally, the still time of the larvae exposed to 100 and 500 μg/L significantly increased (Figure 1L, *p* < 0.05), exhibiting increases of 16.64% and 11.64% compared to the control group, respectively.

### 3.3. Effects of TBBPA-DHEE on the Development of Specific Target Organs in Transgenic Zebrafish

**Figure 2:**
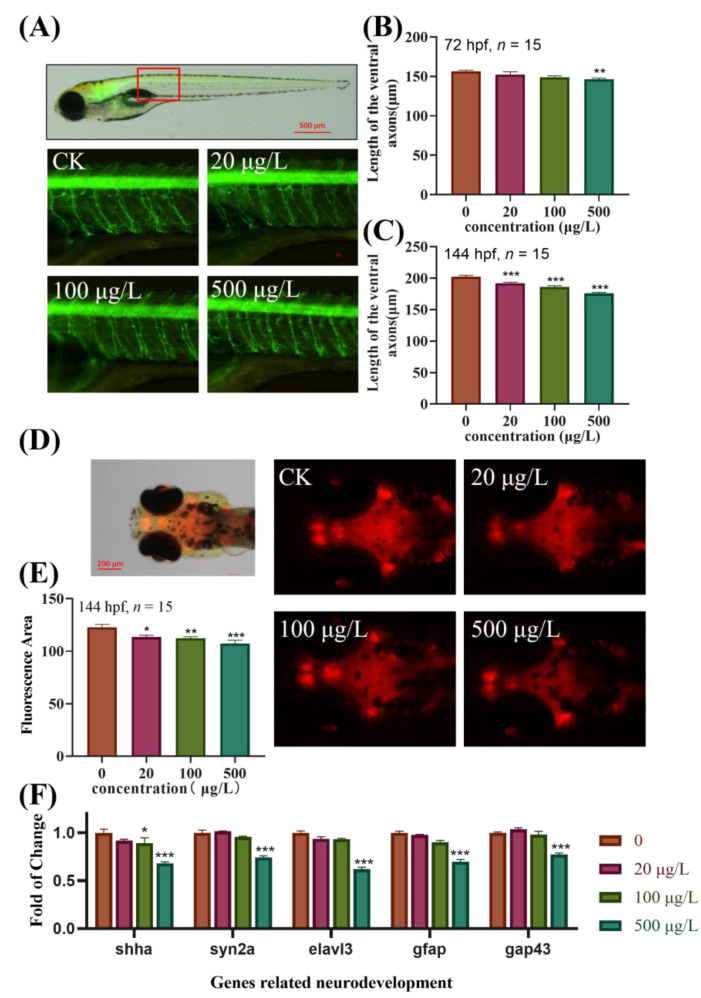
TBBPA-DHEE exposure hampered the development of neurons in zebrafish larvae. (**A**) The typical imagine of *Tg (Hb9: eGFP)* transgenic zebrafish following TBBPA-DHEE exposure; (**B**,**C**) The statistical graph of motor neuron length in zebrafish larvae at 72 and 144 hpf; (**D**) The typical imagine of *Tg (Gad1b: mCherry)* transgenic zebrafish following TBBPA-DHEE exposure; (**E**) The statistical chart of the GAD67-positive GABAergic neurons’ fluorescence area; (**F**) The impact of TBBPA-DHEE on the transcription of genes associated to neurodevelopment, including *shha*, *syn2a*, *elval3*, *gfap* and *gap43*. (* *p* < 0.05, ** *p* < 0.01 and *** *p* < 0.001).

To elucidate the effects of TBBPA-DHEE exposure on the neurodevelopment of zebrafish larvae, transgenic zebrafish strains *Tg (Hb9: eGFP)* and *Tg (Gad1b: mCherry)* were used to further investigate the effects of TBBPA-DHEE on the motor nerve and central nervous system. As shown in Figure 2, in comparison to the control group, after 72 h of exposure to TBBPA-DHEE, the length of the motor neuron axons was shortened, with a significant difference observed solely in the 500 μg/L exposure group (Figure 2B, *p* < 0.01). Notably, after 144 hpf, the lengths of the motor neuron axons in all exposure groups were significantly shortened, being 94.46%, 92.09%, and 86.89% of the control group, respectively (Figure 2C, *p* < 0.001). Additionally, exposure to TBBPA-DHEE impacted on the central nervous system of zebrafish, manifested as a notable decrease in GABAergic neurons in *Tg (Gad1b: mCherry)* transgenic zebrafish, with a concentration-dependent effect detected (Figure 2E, *p* < 0.05).

Based on the above phenotypes, the expression of genes related to neurodevelopment was detected in 144 hpf zebrafish exposed to TBBPA-DHEE. As shown in Figure 2F, compared with the control group, after exposure to 500 μg/L TBBPA-DHEE, the expression levels of neurodevelopment-related genes (*shha*, *syn2a*, *elavl3*, *gfap* and *gap43*) in zebrafish larvae exhibited a significant downward trend (*p* < 0.001), indicating that TBBPA-DHEE can affect the neurodevelopment of zebrafish larvae, verifying the phenotypic results of the aforementioned transgenic zebrafish.

### 3.4. Oxidative Stress Is Induced in Zebrafish Embryos Following Exposure to TBBPA-DHEE

**Figure 3:**
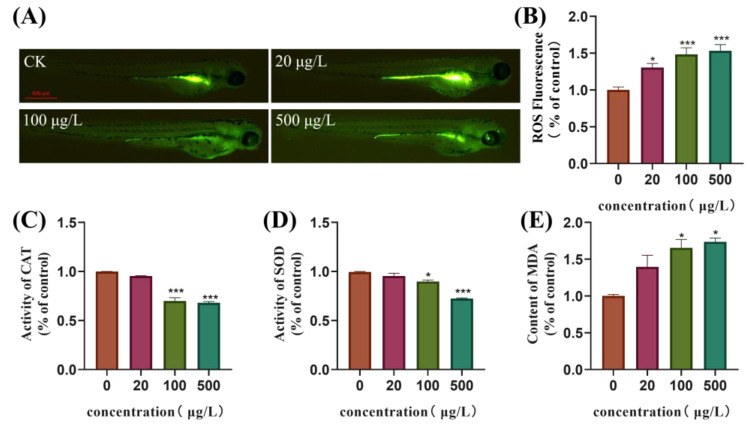
TBBPA-DHEE induces oxidative stress. (**A**) The typical fluorescence imagines of ROS in zebrafish larvae using the DCFH-DA fluorescence probe in difference treatment groups; (**B**) The statistical graph of ROS fluorescence; (**C**–**E**) The activity of CAT, SOD and the MDA content in 144 hpf zebrafish larvae (*n* = 50 per group, * *p* < 0.05 and *** *p* < 0.001).

The content of ROS in zebrafish larvae at 144 h was detected using the DCFH-DA fluorescence probe. The results showed that following exposure to TBBPA-DHEE, the levels of ROS in the larvae of all exposure groups rose significantly in a dose-dependent manner. The fluorescence intensity of zebrafish larvae in the 500 μg/L exposure group was around a factor of 1.5 than that of the control group (Figure 3B, *p* < 0.05). Additionally, the activities of CAT and SOD were suppressed, while the content of MDA rose. Specifically, the activities of CAT and SOD in the 500 μg/L exposure group was about 68.03% and 72.47% of those in the control group, and the content of MDA was 73.72% higher than that in the control group (Figure 3C–E). Furthermore, significant differences were observed in both the medium and high concentration groups (*p* < 0.05).

### 3.5. TBBPA-DHEE Affects the Brain Transcriptome of Juvenile Zebrafish

**Figure 4:**
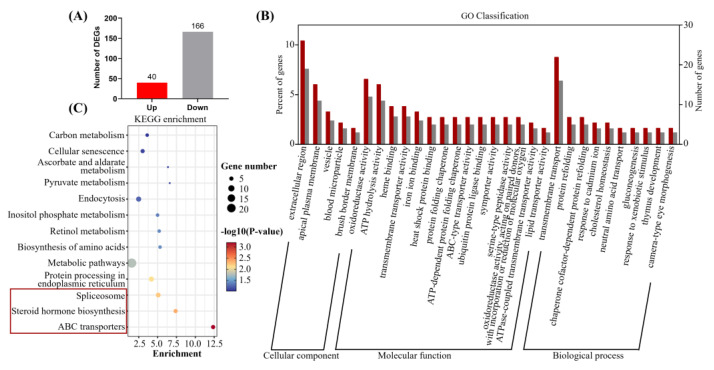
Transcriptome analysis to elucidate the toxicity of neuron development after TBBPA-DHEE exposure. (**A**) The number of DEGs up- and down-regulated between TBBPA-DHEE exposure treatments; (**B**) The top 30 GO terms. (**C**) The KEGG enrichment pathways, Spliceosome, Steroid hormone biosynthesis and ABC transporters were the most significantly enriched pathways.

To further elucidate the potential toxic mechanism of TBBPA-DHEE on zebrafish, zebrafish exposed to 50 μg/L TBBPA-DHEE at 6 hpf were subjected to transcriptome sequencing analysis to assess gene expression during early development. As shown in Figure 4A, compared with the control group, a total of 206 DEGs were identified in the TBBPA-DHEE exposure group (166 down-regulated and 40 up-regulated). The results of GO enrichment analysis based on DEGs revealed the involvement of 29 biological processes, 19 molecular functions, and ten cellular components (Figure 4B). The top ten GO terms with the highest enrichment were primarily focused on biological processes, including protein renaturation, chaperone cofactor-dependent protein renaturation, response to chromium ions, neutral amino acid transport, cardioblast migration to the midline, participation in heart field formation, and establishment of apical/basal polarity in epithelial cells. The KEGG enrichment analysis of DEGs indicated that, in comparison to the control group, TBBPA-DHEE exposure resulting in significant enrichment of DEGs in the ABC transporters, steroid hormone biosynthesis, and spliceosome pathways (Figure 4C). These pathways play crucial roles in neural development and neuroprotection. The above results suggest a possible mechanism of neurotoxicity in zebrafish larvae induced by TBBPA-DHEE.

## 4. Discussion

Several studies have demonstrated the neurotoxic effects of TBBPA in various animal models [23]. TBBPA-DHEE, a derivative of TBBPA, is extensively utilized as a flame retardant in industrial and consumer products. It shares a similar structure to TBBPA and is ubiquitous in the environment [24]. Current research indicates that TBBPA-DHEE disrupts the neuroendocrine function of the hypothalamic-pituitary-gonadal axis in adult zebrafish, leading to reproductive toxicity after prolonged exposure [16]. Furthermore, it causes a decline in motor coordination ability and neurodevelopmental damage in SD rats [14,25]. Studies have indicated that among all the derivatives of TBBPA, TBBPA-DHEE exhibits greater neurotoxicity [17]. However, a systematic study on the early neurotoxic effects and mechanisms of TBBPA-DHEE on aquatic organisms has not yet been conducted. Zebrafish, as a model organism, is often utilized to investigate the ecotoxicity induced by environmental pollutants due to its genomic similarity to humans and analogous brain structure and function [26]. This study utilized zebrafish as a model organism to investigate the neurotoxicity of TBBPA-DHEE. Our study investigated the impacts of TBBPA-DHEE on the early neural development and locomotor behavior of zebrafish, integrated the analysis of relevant gene transcription levels and the assessment of oxidative stress levels, and revealed the potential molecular pathways responsible for these adverse effects via transcriptomics. This systematic investigation aims to clarify the developmental neurotoxicity and the potential mechanisms underlying TBBPA-DHEE-mediated neurotoxicity.

The developmental morphological changes in zebrafish embryos during exposure can most directly reflect the toxic effects of pollutants [27]. In this study, the corresponding developmental indicators were monitored at various time points within the 6 hpf of zebrafish embryos exposed to TBBPA-DHEE. Exposure to TBBPA-DHEE resulting in an elevated death rate of zebrafish embryos, with a 96 hpf LC_50_ of 1.573 mg/L. A concentration of 100 μg/L of TBBPA-DHEE decreased the heart rate of zebrafish larvae, while 500 μg/L of TBBPA-DHEE induced yolk sac edema in zebrafish embryos. Additionally, our study demonstrated that after continuous exposure for 144 hpf, the swim bladder expansion was affected in all exposed groups, and the body length of larvae exposed to 500 μg/L of TBBPA-DHEE was significantly reduced. These findings are consistent with previous research on TBBPA. Wu et al. reported that TBBPA exposure elevated the death rate of zebrafish embryos, resulting in pericardial edema and reduced body length [28]. These findings further emphasize the necessity of investigating the potential neurotoxic effects of TBBPA-DHEE.

The central nervous system is highly susceptible to environmental disturbances during its development [29]. Motor behavior tests have demonstrated efficacy in identifying the neurotoxicity of pharmaceuticals and environmental toxins [30]. In this study, the neurotoxic effects of TBBPA-DHEE were assessed by examining the locomotor behavior of zebrafish larvae exposed to various concentrations of TBBPA-DHEE for 144 hpf. The results indicated that, relative to the control group, exposure to TBBPA-DHEE reduced the locomotion distance and velocity of zebrafish larvae, prolonged the dwell time, and altered the response to the conversion of light and dark light sources. These findings are consistent with those reported by Okeke et al. [31]. Their study revealed that at an exposure concentration of 300 μg/L, there was a downregulation of locomotor behavior-related indicators in zebrafish larvae, whereas at 1500 μg/L, an opposite trend was observed. The reason for this phenomenon remains ambiguous. However, our experimental results demonstrate that TBBPA-DHEE can inhibit the locomotor behavior of zebrafish larvae, which is a signal that triggers neurotoxicity.

In this study, transgenic zebrafish strains *Hb9-eGFP* and *Gad1b-mCherry* were employed to investigate the effects of TBBPA-DHEE on the axons of motor neurons and the development of the central nervous system. The results indicated that exposure to TBBPA-DHEE led to a significant shortening of the axons of motor neurons in zebrafish larvae. In *Tg (Hb9-eGFP)* zebrafish, GFP is specifically expressed in the motor neurons regulated by the hb9 gene, reflecting the development of the motor nerves in the early stage of zebrafish development [32]. Following exposure to TBBPA-DHEE, the length of GFP-labeled motor nerve axons was significantly shortened. The study by Gu et al. reported that exposure to BPAF adversely affected the length of motor neuron axons in zebrafish [33]. Chen et al. found that exposure to TBBPA impeded the development of primary motor neurons in zebrafish [34]. These findings align with the conclusions of this investigation. In *Tg (Gad1b: mCherry)* zebrafish, mCherry-labeled GABAergic neurons are specifically expressed in the central nervous system of the brain, reflecting the development of this system in zebrafish [35]. The results of this study indicated that exposure to TBBPA-DHEE dramatically diminished the fluorescence intensity of mCherry-labeled neurons in the zebrafish brain. Previous studies have shown that exposure to TBBPA can inhibit the development of the central nervous system in zebrafish [36]. These results suggest that TBBPA-DHEE directly impacts the development of the central nervous system and the growth of motor neuron axons.

To elucidate the molecular mechanism by which TBBPA-DHEE induces neurotoxicity in zebrafish, we analyzed the expression levels of several key genes involved in early neural development, including *shha*, *syn2a*, *elavl3*, *gfap* and *gap43*. The study by Zhu et al. demonstrated that TBBPA can lead to a downregulation of the transcriptional level of the *shha* gene, consequently impacting central nervous system development [36]. The member of the synaptophysin family, *syn2a*, plays a crucial role in neurotransmitter release and synaptic formation [37]. The study by Zhang et al. found that a significant downregulation of the *syn2a* gene may induce neurotoxicity by blocking neurotransmitter release [38]. The *elavl3* and *gap43* genes are key components in synapse formation and axonogenesis [39]. The study by Eric et al. found that changes in the *elavl3* and *gap43* genes contribute to the neurodevelopmental toxicity induced by BPA and BPS [40]. The *gfap* gene is expressed in ependymal cells and astrocytes, playing a vital function in the nervous system [41]. In this work, TBBPA-DHEE markedly reduced the expression levels of the *shha*, *syn2a*, *elavl3*, *gfap* and *gap43* genes, further confirming that TBBPA-DHEE induces neurotoxicity in zebrafish larvae.

Oxidative stress is the initial response of organisms to environmental stressors and a key factor in inducing neurotoxicity. Previous studies have shown that exposure to TBBPA in zebrafish leads to a decrease in antioxidant enzyme activity in the central nervous system, resulting in an imbalance of redox homeostasis [28]. The mechanism by which TBBPA-DHEE induces neurotoxicity in zebrafish may be similar to that of TBBPA. Therefore, we assessed the ROS levels and antioxidant enzyme activities in zebrafish larvae exposed to TBBPA-DHEE at 144 hpf. Our research results indicate that exposure to TBBPA-DHEE increases ROS and MDA levels in zebrafish larvae, while reducing the activities of two antioxidant enzymes, SOD and CAT. ROS is a product of redox reactions, and its excessive accumulation can lead to an imbalance in the antioxidant system and induce oxidative stress [42]. MDA is a byproduct of lipid peroxidation in organisms [43]. Under normal conditions, the MDA content is low, but when there is an excessive amount of ROS, macromolecular substances on the biological membrane generate MDA through lipid peroxidation [44,45]. SOD serves as the primary defense against ROS and is the most prevalent antioxidant enzyme in animals [46]. CAT, as one of the most adaptable antioxidant enzymes, is essential for cellular protection against oxidative damage [47,48]. Our research findings indicate that exposure to TBBPA-DHEE in zebrafish damages the antioxidant defense system, thereby causing oxidative stress. This finding aligns with the results of Liu et al., who found that exposure to TBBPA-DHEE induces oxidative stress in PC12 cells, leading to cellular dysfunction [13]. These results further confirm that the oxidative stress pathway contributes to the neurotoxicity elicited by TBBPA-DHEE in zebrafish.

The transcriptome results indicate that, compared to the control group, the DEGs in zebrafish exposed to 500 μg/L TBBPA-DHEE are significantly enriched in pathways related to ABC transporters, steroid hormone biosynthesis, and spliceosome. ABC transporters play a crucial role in numerous neuropathologies [49]. On one hand, they reduce the efficacy of drugs as many therapeutic agents are substrates of ABC transporters [49]. On the other hand, numerous studies have confirmed the function of ABC transporters in various central nervous system diseases [50,51]. Studies have shown that TBBPA alters the function of ABC transporters in zebrafish, hindering drug delivery and increasing the brain’s exposure to exogenous toxins [52]. Cannon et al. demonstrated that GenX inhibits the activity of ABC transporters, thereby interfering with the protective function of the blood-brain barrier (BBB) on the brain [53]. Steroid hormone biosynthesis refers to the enzymatic process by which cholesterol is converted into biologically active steroid hormones [54]. Steroid hormones exert pleiotropic effects on numerous target organs and are of significant importance in neurogenesis and neuroprotection [54]. Lu et al. studied and demonstrated that co-exposure to PFOS and BaP ultimately leads to neurotoxicity by inducing disorders in steroid hormone synthesis [55]. The spliceosome is a crucial component in gene expression and regulation [56]. The sensitivity of neurons to splicing alterations is primarily due to the complexity neuronal cell types and functions in the nervous system, which necessitates specific splicing subtypes to maintain cellular homeostasis [57]. Several studies have revealed the role of splicing regulators in affecting brain development and neuronal functions through gene knockout and transgenic mice [58,59,60]. Our study provides a novel perspective for exploring the molecular mechanism of neurotoxicity induced by TBBPA-DHEE.

## 5. Conclusions

Overall, the study employed a zebrafish model to assess the neurodevelopmental toxicity of TBBPA-DHEE. The findings revealed that TBBPA-DHEE could induce neurotoxicity in zebrafish via the regulation of oxidative stress and neurodevelopmental gene expression. Exposure to TBBPA-DHEE led to developmental deficits in the central nervous system and motor neuron axons of zebrafish, thereby resulting in abnormal motor behaviors. Our study also proposes a novel possible molecular mechanism for TBBPA-DHEE’s neurotoxicity, revealing that TBBPA-DHEE may be a neurotoxic hazard. However, considering the actual environmental levels, the ecological risk of TBBPA-DHEE is relatively limited. With the increasing use of TBBPA-DHEE as a flame retardant, the possible adverse effects it may have on aquatic organisms and public health should be concerned. A limitation of this study is that the toxic targets have not been deeply explored.

## Data Availability

The raw data supporting the conclusions of this article will be made available by the authors on request.

## Supplementary Materials

The following supporting information can be downloaded at: https://www.mdpi.com/article/10.3390/toxics13020076/s1, Table S1: Sequences of primes for the tested genes.
