# Supplementary material for: Neurotoxicity and Mechanism in Zebrafish Embryo Induced by Tetrabromobisphenol A bis (2-Hydroxyethyl) Ether (TBBPA-DHEE) Exposure
Source: Toxics. 2025 Jan 22;13(2):76. doi: 10.3390/toxics13020076 (PMC11860782; doi:10.3390/toxics13020076)
Supplement: Supplementary file 1 [file toxics-13-00076-s001.zip › toxics-3443812-supplementary.pdf]

toxics MDPI

Table S1. Primer Sequences for the tested genes.

Name	Gene no.	Primer sequence
β-actin	NM_181601.5	Forward: 5′-TCGAGCAGGAGATGGGAACC-3′<br>Reverse: 5′-CTCGTGGATACCGCAAGATTC-3′
shha	NM_181601.5	Forward: 5′-AGACCGAGACTCCACGACGC-3′<br>Reverse: 5′-TGCAGTCACTGGTGCGAACG-3′
syn2a	NM_001002597.2	Forward: 5′-GTGACCATGCCAGCATTTC-3′<br>Reverse: 5′-TGGTTCTCCACTTTCACCTT-3′
elval3	NM_131449.1	Forward: 5′-AGACAAGATCACAGGCCAGAGCTT-3′<br>Reverse: 5′-TGGTCTGCAGTTTGAGACCGTTGA-3′
gfap	XM_005163983.4	Forward: 5′-GGATGCAGCCAATCGTAAT-3′<br>Reverse: 5′-TTCCAGGTCACAGGTCAG-3′
gap43	NM_131341.2	Reverse: 5′-TTCCAGGTCACAGGTCAG-3′<br>Reverse: 5′-CCTCCGGTTTGATTCCATC-3′
